# Circulating Tumor DNA in Upper Tract Urothelial Carcinoma: A Framework for Precision Perioperative Management

**DOI:** 10.3390/cancers18101651

**Published:** 2026-05-20

**Authors:** Amulya Prakash, Adriani Cherico, Adanma Ayanambakkam, Hyma Vani Polimera

**Affiliations:** 1 Penn State Cancer Institute, 400 University Dr., Hershey, PA 17033, USA; aprakash1@pennstatehealth.psu.edu (A.P.); acherico@pennstatehealth.psu.edu (A.C.); 2 Department of Hematology-Oncology, Penn State College of Medicine, 700 HMC Crescent Rd., Hershey, PA 17033, USA; 3 Stephenson Cancer Center, University of Oklahoma Health Sciences Center, Oklahoma City, OK 73104, USA; adanma-ayanambakkam@ou.edu

**Keywords:** upper tract urothelial carcinoma, circulating tumor DNA, urinary tumor DNA, molecular residual disease, bladder cancer, perioperative management

## Abstract

Upper tract urothelial carcinoma (UTUC) presents distinct diagnostic and therapeutic challenges because of its rarity, anatomic constraints, frequent understaging at biopsy, and risk of systemic recurrence after radical nephroureterectomy. Current perioperative management is driven primarily by clinicopathologic risk factors, which may be insufficient to identify occult molecular residual disease (MRD) or to determine which patients are most likely to benefit from systemic therapy. This narrative review summarizes available evidence on circulating tumor DNA (ctDNA) in UTUC and related urothelial carcinoma settings, classifies the level of evidence supporting each application, and proposes a research framework for prospective evaluation. The strongest UTUC-specific evidence supports ctDNA as a prognostic biomarker associated with recurrence risk, whereas predictive validity for selecting chemotherapy, immune checkpoint inhibitors, antibody-drug conjugates, targeted therapy, or surveillance intensity remains unproven. Evidence from muscle-invasive bladder cancer, including ctDNA-correlative and ctDNA-guided perioperative trials, provides biologic rationale but should not be directly translated into routine UTUC care without disease-specific validation. We outline key implementation questions, including target population, assay selection, timing, false-positive and false-negative results, lead-time bias, and integration of plasma ctDNA with utDNA. Prospective UTUC-specific trials are needed to determine whether ctDNA-guided perioperative strategies improve survival, reduce unnecessary toxicity, and are cost-effective.

## Simple Summary

Upper tract urothelial carcinoma (UTUC) is a rare cancer of the renal pelvis and ureter that is difficult to diagnose, stage accurately, and treat, with many patients experiencing recurrence even after surgery. Current treatment decisions rely largely on clinical and pathological features that are often insufficient. Circulating tumor DNA (ctDNA) is an emerging biomarker that can detect molecular residual disease and may identify patients at higher risk of recurrence, enabling more personalized treatment. However, UTUC-specific evidence remains limited, and most available data are extrapolated from bladder cancer or broader urothelial carcinoma studies. In this narrative review, we synthesize the current evidence and propose a research framework for integrating ctDNA in UTUC perioperative management. We emphasize that ctDNA-guided treatment escalation, de-escalation, and surveillance modification remain investigational and require prospective UTUC-focused validation before routine clinical implementation.

## 1. Introduction

Upper tract urothelial carcinoma (UTUC), involving the ureter and renal pelvis, accounts for approximately 5–10% of all urothelial cancers (UC) [1]. The management of UTUC presents unique challenges. While transurethral resection of bladder tumor (TURBT) is the diagnostic standard for UC of the bladder, ureteroscopic biopsy in UTUC is often limited by tumor location, small biopsy volume, cautery artifact, and the thin-walled anatomy of the upper tract. Ureteroscopic tumor biopsy understages UTUC in up to 46% of the cases [2].

Current risk stratification relies heavily on tumor grade, radiographic findings, hydronephrosis, multifocality, lymphovascular invasion, pathologic stage, and nodal status. These variables are clinically useful but imperfect surrogates for occult systemic disease. Current clinicopathologic models may therefore inadequately identify micrometastatic disease and unreliably predict which patients are most likely to benefit from perioperative systemic therapy. As a result, perioperative systemic therapy may overtreat some patients while failing to prevent recurrence in others. Despite curative-intent radical nephroureterectomy (RNU) for localized high-risk disease, recurrence rate remains high, particularly among patients with ≥pT2 disease, lymphovascular invasion, positive margins, or nodal involvement [3]. This clinical ambiguity necessitates the development of reliable biomarkers for prognostication and treatment stratification.

Circulating tumor DNA (ctDNA) is a non-invasive biomarker that may detect molecular residual disease (MRD) after definitive local therapy. In urothelial carcinoma, ctDNA has shown prognostic value in bladder cancer and in limited UTUC-specific cohorts; however, the evidence base in UTUC remains substantially less mature. Importantly, prognostic value should be distinguished from predictive value. A prognostic biomarker identifies patients at higher risk of recurrence, whereas a predictive biomarker identifies patients more likely to benefit from a specific treatment. In UTUC, ctDNA is best viewed at present as a promising prognostic tool and investigational predictive biomarker.

**Figure 1:**
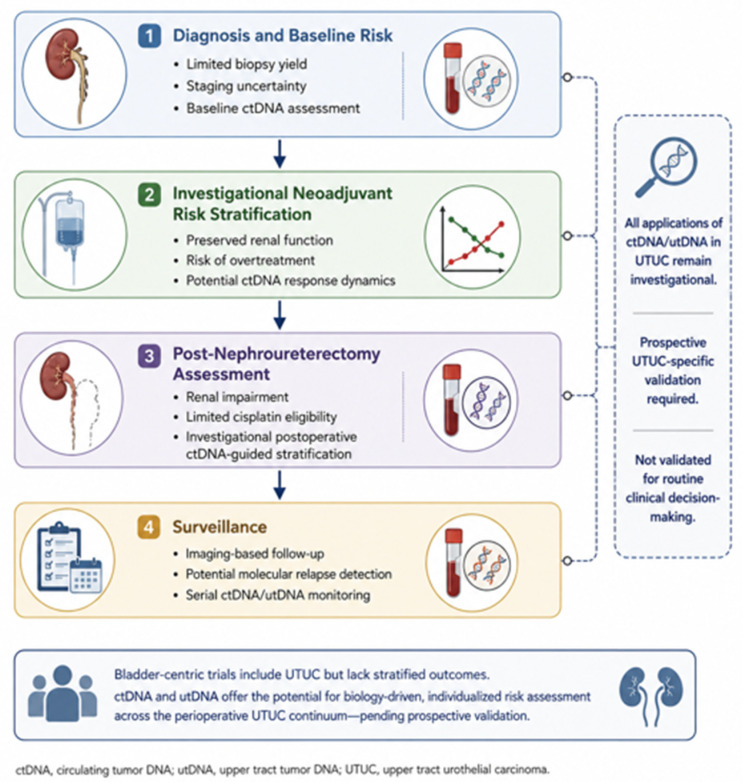
Conceptual investigational framework for ctDNA integration in upper tract urothelial carcinoma.

This narrative review synthesizes the current evidence and proposes a disease-specific research framework for evaluating ctDNA and utDNA across the perioperative UTUC continuum. The proposed framework is not intended as a clinical algorithm and should not be used to guide routine treatment escalation, de-escalation, or surveillance modification outside prospective studies. A conceptual overview of investigational ctDNA integration across the perioperative UTUC continuum is illustrated in Figure 1.

## 2. Methods

This article is a narrative review rather than a systematic review. A structured literature search was performed to identify relevant studies evaluating ctDNA, utDNA, MRD, perioperative systemic therapy, and clinical trials in UTUC and urothelial carcinoma. PubMed/MEDLINE, ClinicalTrials.gov, and major oncology meeting abstracts were searched from January 2010 through May 2026. Search terms included combinations of: “upper tract urothelial carcinoma,” “UTUC,” “urothelial carcinoma,” “bladder cancer,” “circulating tumor DNA,” “ctDNA,” “molecular residual disease,” “MRD,” “urine tumor DNA,” “utDNA,” “perioperative,” “neoadjuvant,” “adjuvant,” “immunotherapy,” “enfortumab vedotin,” “antibody-drug conjugate,” and “surveillance.”

Priority was given to prospective trials, randomized studies, UTUC-specific ctDNA studies, urothelial carcinoma studies that included UTUC patients, and high-quality translational studies relevant to MRD detection. Studies were excluded if they did not address urothelial carcinoma, did not include perioperative or MRD-relevant biomarker data, or were not available in English. Because this was not a systematic review, formal risk-of-bias assessment and meta-analysis were not performed. Evidence was categorized as UTUC-specific, bladder cancer-specific, pan-urothelial/unstratified, or hypothesis-generating extrapolation.

## 3. Current Treatment Landscape in Upper Tract Urothelial Cancer

UTUC remains underrepresented in prospective perioperative clinical trials, and many treatment recommendations continue to be extrapolated from bladder cancer data. Radical nephroureterectomy (RNU) with complete bladder cuff excision remains the foundation of curative-intent management for high-risk disease, whereas kidney-sparing approaches are generally reserved for carefully selected low-risk tumors [4,5]. The phase III POUT trial established adjuvant platinum-based chemotherapy as an evidence-based perioperative treatment option for patients with resected high-risk UTUC. In patients with ≥pT2 or node-positive disease following RNU, adjuvant gemcitabine plus cisplatin or carboplatin significantly improved disease-free survival compared with surveillance [4].

Neoadjuvant cisplatin-based chemotherapy is increasingly considered for cisplatin-eligible patients with high-risk UTUC because renal function frequently declines after RNU, potentially limiting postoperative cisplatin eligibility. However, this approach is not supported by the same level of randomized, UTUC-specific evidence as adjuvant chemotherapy and may expose some patients to treatment-related toxicity or surgical delay without clear benefit. The iNDUCT trial is among the few prospective studies specifically evaluating neoadjuvant chemoimmunotherapy in localized UTUC. In this phase II study, neoadjuvant durvalumab combined with platinum-based chemotherapy demonstrated a pathologic complete response rate of approximately 19–21% and pathologic downstaging (<ypT2N0) in nearly 60% of patients, while more than 85% of patients successfully proceeded to surgery with preservation of acceptable renal function [6]. Grade ≥ 3 adverse events occurred in approximately 35–40% of patients without unexpected safety signals. Although the trial did not meet its primary endpoint of pathologic complete response, the observed tumor downstaging and acceptable safety profile suggest feasibility and potential clinical activity. Nevertheless, these studies remain relatively small and require prospective validation in larger, adequately powered UTUC-specific trials before altering standard clinical practice.

Recent perioperative and metastatic urothelial carcinoma trials have transformed the therapeutic landscape, particularly with immune checkpoint inhibitors and antibody–drug conjugates (ADCs). However, many of these studies were bladder cancer-centric, excluded UTUC, or included UTUC patients without reporting disease-specific subgroup outcomes. Therefore, the applicability of these findings to UTUC should be interpreted cautiously and largely considered indirect unless dedicated upper tract analyses are available. As summarized in Table 1, although patients with UTUC were included in several landmark urothelial carcinoma trials, stratified efficacy outcomes specific to upper tract disease were generally not reported. This highlights the current reliance on extrapolated evidence and underscores the need for prospective biomarker-driven and molecularly informed treatment strategies tailored specifically to UTUC.

UTUC differs from bladder urothelial carcinoma not only anatomically but also at the molecular and histologic levels. Anatomically, the thin muscularis layer of the upper tract and early access to lymphovascular channels contribute to a higher propensity for systemic dissemination even in apparently localized disease. Histologically, although both arise from urothelium, UTUC demonstrates distinct genomic features, with higher frequencies of FGFR3, KMT2D, and MYC alterations, whereas bladder tumors more commonly harbor TP53, RB1, TERT, and ERBB2 mutations [7]. UTUC has a strong association with Lynch syndrome and tends to have higher rates of microsatellite instability and DNA mismatch repair (MMR) deficiency than bladder cancer, which poses therapeutic implications [8]. UTUC typically shows lower levels of PD-L1 expression and tumor mutational burden than bladder cancer, both of which are generally associated with weaker responses to immunotherapy [7]. However, UTUC patients with Lynch syndrome may be better candidates for immunotherapy due to the presence of microsatellite instability and MMR deficiency. These differences highlight the need for a more individualized approach to the management of UTUC, with a specific focus on genetics, molecular biomarkers, and immunologic profiling.

## 4. UTUC-Specific Evidence for ctDNA and utDNA

Given these biologic and molecular differences, it is important to distinguish evidence derived specifically from UTUC cohorts from findings extrapolated from bladder cancer or pan-urothelial carcinoma studies. The available UTUC-specific ctDNA literature remains limited but provides an important foundation. The strongest signal thus far is prognostic, with detectable perioperative ctDNA associated with adverse pathologic features and recurrence risk. However, no prospective UTUC-specific trial has yet demonstrated that ctDNA-guided therapeutic intervention improves survival outcomes. Table 2 summarizes the currently available ctDNA and utDNA evidence relevant to UTUC, including ongoing investigational studies, and distinguishes direct evidence from extrapolation.

Collectively, the currently available literature supports the prognostic relevance of ctDNA in urothelial carcinoma and suggests potential applicability in UTUC; however, prospective UTUC-specific validation of ctDNA-guided treatment strategies remains lacking.

CtDNA may offer a biologically informed strategy to refine perioperative decision-making in UTUC beyond traditional clinicopathologic risk stratification. Rather than applying treatment algorithms derived from bladder cancer trials, it is plausible that ctDNA-informed strategies could offer a more nuanced approach. Such a framework might help identify patients with biologically aggressive UTUC who are more likely to derive benefit from adjuvant therapy, while potentially sparing those at lower risk of recurrence from the burdens of unnecessary toxicity. The prognostic relevance of ctDNA has been demonstrated in MIBC. The post hoc analysis from IMvigor010 trial revealed a strong correlation between ctDNA status and treatment benefit. Patients who were ctDNA positive after surgery had a significantly higher rate of recurrence and benefited from adjuvant atezolizumab treatment [11]. On the other hand, ctDNA negative patients demonstrated no benefit with adjuvant atezolizumab compared to observation. Building on these observations, the IMvigor011 was designed as a prospective, ctDNA-guided trial in which ctDNA-positive patients were randomized to adjuvant immunotherapy versus placebo. For ctDNA-positive patients with detectable MRD, atezolizumab almost doubled DFS (9.9 vs. 4.8 months) compared to placebo and reduced the risk of disease recurrence by 36% in this population [12]. The NIAGARA and CheckMate274 trials added further to the expanding role of perioperative immune checkpoint inhibitor therapy and raised critical questions regarding patient selection [13,14].

Recent advances in systemic therapy have transformed outcomes in UC. Enfortumab vedotin (EV), a nectin-4-directed antibody–drug conjugate (ADC), particularly when combined with PD-1 inhibitors, has demonstrated superiority over traditional chemotherapy regardless of cisplatin eligibility. In the metastatic setting, the KEYNOTE-A39/EV-302 trial demonstrated that EV plus pembrolizumab was the first chemotherapy-free regimen to significantly improve overall survival compared with platinum-based chemotherapy in previously untreated advanced urothelial carcinoma, irrespective of cisplatin eligibility, thereby establishing a new global standard of care [15]. These findings supported the evaluation of this approach in localized disease in phase III KEYNOTE-905/EV-303 trial. In cisplatin-ineligible patients with MIBC, perioperative EV plus pembrolizumab reduced the risk of recurrence, progression, or death by 60% compared with surgery alone (EFS HR: 0.40; *p* < 0.0001) and reduced the risk of death by 50% (OS HR: 0.50; *p* = 0.0002) [16]. More recently, the phase III KEYNOTE-B15/EV-304 trial evaluated perioperative EV plus pembrolizumab versus standard neoadjuvant gemcitabine–cisplatin chemotherapy in patients with cisplatin-eligible muscle-invasive bladder cancer undergoing radical cystectomy. The combination significantly improved event-free survival (EFS), reducing the risk of recurrence, progression, or death by 47% (HR: 0.53) compared with standard chemotherapy, with a 2-year EFS rate of 79.4% versus 66.2%, respectively [17]. The regimen also demonstrated improved overall survival and higher pathologic complete response rates (≈55.8% vs. 32.5%), suggesting that perioperative EV plus pembrolizumab may represent a new potential standard of care in this setting. These results, which led to the FDA approval of this regimen in November 2025, establish the ADC plus PD-1 combination therapy as a new benchmark in modern urothelial cancer therapy. Importantly, the previously mentioned landmark trials did not specifically focus on upper tract disease; although UTUC patients were included in most of these studies, stratified analyses were not reported.

In UTUC management, a critical gap persists due to the absence of robust biomarkers capable of identifying occult systemic disease beyond histopathologic assessment. CtDNA may serve as a potential marker of molecular residual disease and may eventually support more individualized, biomarker-informed management strategies (Table 3). We hypothesize a conceptual framework where detection of MRD after surgery could identify patients most likely to benefit from adjuvant systemic therapy, while ctDNA-negative patients may represent a lower-risk population for prospective de-escalation strategies.

Under this theoretical model, preoperative ctDNA positivity could help select patients for intensified neoadjuvant approaches before postoperative renal function decline limits treatment options. Furthermore, persistent ctDNA despite systemic therapy may identify patients suitable for prospective evaluation of earlier surgical intervention or modification of systemic agents. If validated through prospective trials, this real-time monitoring could address a central concern in UTUC management: disease progression during the neoadjuvant window. While CheckMate274 established the efficacy of immunotherapy in post-operative MIBC, its results support moving immunotherapy earlier in the treatment sequence in a more selective manner [14]. For selected patients, ctDNA positivity could identify candidates for prospective evaluation of chemo-sparing perioperative strategies incorporating EV plus pembrolizumab, particularly in those seeking to avoid platinum-related nephrotoxicity, neuropathy, and myelosuppression [16]. Conversely, ctDNA-negative patients may represent a lower-risk population for prospective evaluation of treatment de-escalation strategies. This approach is particularly relevant for cisplatin-ineligible patients, for whom platinum-based adjuvant therapy is not feasible and for whom immunotherapy or ADC-based regimens may serve as alternatives. Given the modest absolute benefit observed with adjuvant therapy in unselected populations, improved molecular stratification remains essential [18]. The MODERN trial is an ongoing, randomized, multicenter clinical trial investigating the use of ctDNA to guide adjuvant immunotherapy in patients with high-risk muscle-invasive urothelial carcinoma (MIUC) after radical surgery, aimed at validating whether ctDNA can be used as a predictive biomarker to guide adjuvant therapy decisions in UC [19]. The TOMBOLA trial supports ctDNA-guided, risk-adapted management in urothelial carcinoma by using serial testing to trigger early immunotherapy intervention. The study demonstrated high recurrence-free rates among ctDNA-negative patients (97–98%) and identified a median lead time of approximately 90 days between ctDNA detection and radiographic relapse in ctDNA-positive patients [20]. Collectively, these findings reflect an investigational shift toward a more individualized management approach that incorporates molecular risk assessment alongside traditional clinicopathologic staging.

To further conceptualize potential investigational applications of ctDNA and utDNA in UTUC, we propose a simplified research-oriented framework summarizing how molecular residual disease assessment could potentially inform future perioperative and surveillance strategies (Table 3). These concepts are intended to illustrate prospective research directions rather than establish routine clinical decision-making algorithms.

This framework intentionally uses research-conditional language and should not be interpreted as evidence supporting routine ctDNA-guided treatment selection in UTUC.

## 5. Role of CtDNA in UTUC

CtDNA refers to fragmented tumor DNA released into the bloodstream through apoptosis, necrosis, and active secretion [21]. CtDNA assays are categorized into tumor-informed approaches, which track patient-specific somatic mutations identified from the primary tumor, and tumor-agnostic (plasma-only) approaches, which use predefined gene panels to detect common cancer-associated alterations without prior tumor sequencing [22]. CtDNA has emerged as a sensitive biomarker for detecting MRD and occult systemic involvement. CtDNA may offer the potential to move beyond the “one-size-fits-all” approach and toward biologically informed, personalized treatment strategies for UTUC. Preclinical models further highlight the marked genomic heterogeneity and variability in therapeutic response in urothelial carcinoma, underscoring the limitations of static risk stratification and supporting the need for dynamic biomarkers [23,24]. Advances in next-generation sequencing technologies and error-correction methods have enabled highly sensitive detection of ctDNA at variant allele frequencies below 0.1%, allowing for identification of MRD following curative-intent therapy [25]. In UTUC, emerging evidence from recent UC trials suggests that the persistent ctDNA detection following radical surgery is associated with an increased risk of recurrence, frequently identifying molecular residual disease months before radiographic or clinical relapse becomes apparent [12]. This ability to detect molecular relapse before clinical progression positions ctDNA as a prognostic biomarker, while its utility as a predictive tool to guide adjuvant therapy remains investigational.

The clinical integration of ctDNA in stage II colon cancer provides a proof-of-concept for MRD-guided treatment strategies. The most practice-changing evidence comes from the DYNAMIC trial, a prospective randomized study in stage II colon cancer that compared ctDNA-guided management with standard clinicopathologic decision-making [26]. Incorporating ctDNA shifted treatment decisions from anatomy-based risk assessment to biology-driven precision oncology, thereby providing a compelling framework for future ctDNA-guided approaches in urothelial cancers, particularly UTUC. However, caution is warranted when extrapolating these findings to UTUC, as colon cancer is associated with higher ctDNA shedding rates and differs substantially from UTUC in both tumor biology and treatment approaches.

### 5.1. Scope

The application of ctDNA in UTUC offers several theoretical advantages and represents a significant transition from anatomy-based staging towards a dynamic genomic assessment of disease burden. CtDNA assessment following RNU has the potential to serve as a specific marker of micrometastatic disease and residual tumor burden. CtDNA has emerged as a sophisticated tool for MRD detection; however, ongoing investigations are evaluating whether it can complement or potentially replace traditional pathologic risk stratification methods in guiding clinical management [19]. At the same time, it can also detect molecular relapse months before conventional cross-sectional imaging can visualize macro-metastases.

In the current clinical landscape, pathologic risk stratification often fails to capture the true risk of systemic recurrence. Patients who are found to be ctDNA negative postoperatively might safely be spared the morbidity of adjuvant therapy. This is particularly relevant in the UTUC population, where RNU-induced renal insufficiency often complicates the administration of cisplatin-based chemotherapy or increases the risk of treatment-related toxicity in patients with competing comorbidities. Conversely, those patients who remain ctDNA positive following surgery represents a biologically high-risk group in whom adjuvant systemic therapy, including immunotherapy or novel targeted agents, may offer the greatest absolute benefit, pending prospective UTUC-specific validation.

Beyond the postoperative setting, ctDNA could be leveraged to guide neoadjuvant treatment strategies in UTUC. Neoadjuvant chemotherapy is often advocated when renal function is preserved and may improve systemic control. Yet not all patients benefit from this approach, and some may experience unnecessary toxicity or delays in surgery. Baseline ctDNA detection prior to surgery may help identify patients most likely to benefit from perioperative systemic therapy. Furthermore, monitoring ctDNA dynamics during the course of treatment could serve as an early surrogate marker of therapeutic response. The rapid clearance of ctDNA following neoadjuvant therapy may indicate effective eradication of micrometastatic disease, whereas persistent ctDNA could prompt treatment intensification or alternative strategies.

In addition to plasma-based approaches, urine-based tumor DNA (utDNA) represents a complementary modality with particular relevance in UTUC, given the direct shedding of tumor DNA into the urinary tract. Compared with plasma ctDNA, utDNA may offer enhanced sensitivity for detecting localized or low-volume disease. This may be particularly relevant in UTUC following radical nephroureterectomy, where patients remain at substantial risk for subsequent urothelial recurrence within the remnant urinary tract and bladder. In this setting, utDNA could potentially serve as a non-invasive tool for monitoring the remnant urothelium and detecting bladder recurrence earlier than conventional surveillance modalities. Emerging evidence in urothelial carcinoma suggests that utDNA can detect tumor-derived alterations with high specificity and may help distinguish localized from systemic disease [27]. Integration of utDNA with plasma ctDNA could therefore provide a more comprehensive assessment of disease burden by capturing both local and systemic tumor dynamics, although prospective UTUC-specific validation remains necessary [28]. However, challenges related to assay standardization, biological variability, and optimal clinical implementation remain to be addressed.

### 5.2. Limitations

Despite these promising concepts, several significant gaps in knowledge limit the immediate clinical adoption of ctDNA-guided therapy in UTUC. One major challenge relates to the lack of assay standardization and sensitivity. UTUC tumors often have a lower tumor mutational burden compared with bladder cancer, which may affect ctDNA shedding, which directly impacts the concentration of ctDNA in the blood and lowers the detection rates for traditional assays, increasing the risk of false-negative results in low-shedding tumors [29]. This necessitates a careful choice between testing methodologies. Tumor-informed assays, which require sequencing of the primary tumor to track patient-specific variants, offer higher sensitivity but are more resource-intensive and may be logistically challenging in routine practice. Tumor-agnostic assays provide broader applicability but may lack the sensitivity required for MRD detection in early-stage disease. In those cases, a plasma-based deep sequencing approach may be preferable for UTUC, as demonstrated by Nakano et al.; however, the findings were limited by the small cohort size, retrospective design, and lack of standardized ctDNA-guided therapeutic intervention [9,30]. In addition, lack of assay harmonization across platforms, including variability in sensitivity thresholds and reporting standards, further limits comparability and clinical implementation.

Another unresolved issue is the optimal timing and frequency of ctDNA testing. Postoperative ctDNA assessment shortly after nephroureterectomy may be confounded by transient elevations in background cell-free DNA related to surgical trauma and inflammation. Prior studies in solid tumors suggest that immediate postoperative sampling may increase the risk of false-positive interpretation, and several studies in other malignancies have proposed delaying postoperative ctDNA assessment for approximately 2–4 weeks after surgery to improve assay specificity and reduce confounding from surgical DNA release [25]. However, delaying the test risks missing the critical window for adjuvant intervention before the onset of gross metastatic progression. Prospective studies are needed to define the optimal timing, frequency, and longitudinal interpretation of ctDNA testing. Serial monitoring could provide valuable insights into disease dynamics, but the clinical thresholds for intervention remain undefined, limiting interpretation of both prognostic and predictive value. Furthermore, earlier detection of molecular relapse through ctDNA may introduce lead-time bias, whereby recurrence is identified earlier without necessarily improving overall survival, as treatment strategies guided by ctDNA findings remain investigational.

It is important to acknowledge that the clinical implementation of a ctDNA-guided approach must account for the significant oncologic, psychological, and economic risks inherent in assay inaccuracies. A false-negative result where molecular residual disease remains undetected could lead to the omission or delay of life-saving adjuvant therapy, while a false-positive result, potentially driven by clonal hematopoiesis of indeterminate potential, might subject patients to the toxicities of intensive systemic treatments for disease that is not actually present. Beyond these clinical implications, inaccurate ctDNA results may also impose a substantial human burden; a positive ctDNA finding can provoke significant patient anxiety and psychological distress, while the use of costly therapies such as antibody–drug conjugates or immunotherapy may contribute to considerable financial toxicity when treatment is ultimately unnecessary. Given that the long-term impact on quality of life and cost-effectiveness remains unquantified, these factors reinforce that ctDNA-based frameworks should currently be viewed as investigational hypotheses requiring rigorous prospective validation.

The predictive utility of ctDNA for specific therapeutic modalities in UTUC remains unproven. While the data from bladder cancer trials, such as IMvigor010, suggest that ctDNA positivity predicts benefit from immunotherapy in bladder cancer, it is unclear whether the same holds true in upper tract disease, which has distinct molecular characteristics, including a higher prevalence of FGFR3 and HER2 alterations, as well as a significant association with Lynch syndrome and microsatellite instability [7]. CtDNA could potentially serve as a platform for integrating genomic profiling with minimal residual disease detection, enabling the selection of targeted therapies or combination approaches in the adjuvant setting. However, this hypothesis requires validation in dedicated UTUC cohorts. Additionally, cost and access to ctDNA testing remain important barriers, particularly in community and resource-limited settings, which may further exacerbate disparities in care.

## 6. Future Directions

Future clinical trial designs should prioritize the integration of ctDNA as a central, pre-specified component rather than a secondary or exploratory endpoint. In UTUC, where therapeutic decision-making is often constrained by limited evidence and heterogeneous risk profiles, biomarker-driven trials may offer a critical path forward. Clinical trials specifically enrolling ctDNA-positive patients following nephroureterectomy and randomizing them to adjuvant systemic therapy versus observation would provide the most definitive evidence of clinical utility and determine whether MRD-guided intervention improves survival outcomes and other clinically meaningful endpoints. Such designs could potentially establish ctDNA as a predictive biomarker capable of identifying patients most likely to benefit from treatment escalation.

Several ongoing urothelial carcinoma trials are currently evaluating ctDNA-guided therapeutic strategies and may provide important insights relevant to future UTUC-specific trial development. However, dedicated prospective studies specifically designed for UTUC remain limited. Future UTUC-specific studies should prospectively enroll patients with high-risk localized disease, incorporate predefined ctDNA time points, and evaluate paired plasma ctDNA and utDNA approaches. Stratification by renal pelvis versus ureteral primary, Lynch syndrome/MMR status, FGFR3 alterations, renal function, and perioperative systemic therapy may further refine biologic interpretation. Given the rarity of UTUC, adaptive and collaborative trial designs will likely be important, although safeguards addressing false-positive and false-negative ctDNA results remain essential.

Adaptive trial designs could allow escalation or de-escalation of therapy based on ctDNA dynamics, reflecting a more personalized and biology-driven approach to cancer care. For example, patients with persistent ctDNA positivity after initial therapy could be prospectively evaluated for combination regimens or novel agents, whereas those demonstrating early ctDNA clearance might be candidates for treatment de-escalation or abbreviated therapy duration within prospective clinical trials. This approach aligns with a broader shift toward response-adapted oncology, where therapeutic intensity is continuously refined based on evolving tumor biology rather than fixed treatment algorithms. Platform trials incorporating multiple therapeutic arms such as immunotherapy, ADCs, and targeted therapies could be particularly valuable in UTUC, allowing for efficient evaluation of ctDNA-guided strategies across diverse treatment modalities. Importantly, given the relative rarity of UTUC, multi-institutional and international collaboration will be pivotal to achieve adequate patient accrual, increase statistical power, and ensure that findings are generalizable across populations.

Beyond its role in guiding perioperative systemic therapy, ctDNA holds significant promise in reshaping surveillance strategies in UTUC. Current follow-up protocols rely heavily on cross-sectional imaging and invasive procedures such as cystoscopy and ureteroscopy, which are associated with substantial cost, patient burden, and cumulative radiation exposure. A ctDNA-based surveillance approach could enable earlier detection of molecular relapse, potentially preceding radiographic progression by several months. This earlier window of detection may create an opportunity for timely therapeutic intervention at a stage of lower disease burden, where treatment efficacy may be maximized. In addition, serial ctDNA monitoring could allow for risk-adapted surveillance intensity, reducing the frequency of imaging and invasive procedures in patients with persistently negative ctDNA results, thereby improving quality of life while potentially reducing unnecessary interventions. However, the psychological impact of disease surveillance through molecular monitoring and the management of molecular relapse in the absence of radiographic disease must be carefully considered. The identification of MRD or early molecular relapse may generate significant anxiety for patients, particularly when the clinical implications are uncertain. It should be noted that the earlier detection alone is unlikely to translate into a survival benefit unless effective therapeutic interventions are available and initiated at the time of molecular relapse. Therefore, the clinical value of ctDNA-guided surveillance will ultimately depend on whether earlier intervention based on ctDNA positivity can meaningfully alter disease course and improve patient outcomes. Clear communication, shared decision-making, and supportive care frameworks will be essential to ensure that the benefits of molecular monitoring are not offset by unintended psychosocial harm. In addition, the cost-effectiveness of serial ctDNA testing must be evaluated, particularly in comparison with existing surveillance strategies.

The integration of plasma-based ctDNA with utDNA represents another promising avenue for enhancing detection sensitivity and disease localization [31]. Given the anatomic proximity of UTUC tumors to the urinary tract, utDNA may provide a more concentrated and tumor-specific signal, particularly for localized or urothelial-confined disease [28,32]. A combined approach leveraging both plasma and urine assays could improve diagnostic accuracy by distinguishing local recurrence from systemic disease. Future studies should focus on defining the optimal timing, frequency, and clinical context for integrating these modalities into perioperative management algorithms.

## 7. Conclusions

In conclusion, ctDNA represents a promising prognostic biomarker in UTUC and may eventually support more precise perioperative risk stratification and molecularly informed management strategies. However, ctDNA-guided treatment selection, escalation, de-escalation, and surveillance modification remain investigational in UTUC and should not currently be interpreted as validated clinical practice. The existing evidence base is limited by small UTUC-specific cohorts, reliance on extrapolation from bladder cancer studies, lack of assay standardization, uncertain testing intervals, and the absence of prospective UTUC-specific interventional trials. Future studies should prospectively validate both ctDNA and utDNA in clearly defined UTUC populations and determine whether biomarker-guided intervention can meaningfully improve survival outcomes, quality of life, cost-effectiveness, and equity in care delivery. Until such data are available, ctDNA-guided management should be viewed primarily as a research framework rather than a routine clinical decision-making algorithm in UTUC.

## Figures and Tables

**Table 1:** Landmark urothelial carcinoma trials informing perioperative and metastatic management, with UTUC relevance explicitly classified.

Trial	Trial Population (N)	Intervention vs. Comparator	UTUC Included	Relevance to UTUC	Primary Endpoint(s)	Key Efficacy Outcome(s)	UTUC Specific Outcomes	ctDNA Utilized	Hazard Ratio	*p*-Value
POUT	Adults with resected UTUC (261)	Adjuvant chemotherapy vs. surveillance	Yes (100%)	Direct	DFS	Adjuvant platinum chemotherapy improved DFS vs. surveillance	Improvement in DFS with adjuvant chemotherapy	No	DFS HR: 0.45	0.0001
IMvigor 010	Adults with MIBC able to undergo surgery (809)	Adjuvant atezolizumab vs. observation alone	Yes (7%)	Direct	DFS	Negative in ITT—no significant DFS in the study populations	Adjuvant atezolizumab did not improve DFS	Yes (exploratory)	0.89	0.24
IMvigor 011	Adults with MIBC and are disease-free radiologically (761)	ctDNA + patients randomized to receive atezolizumab or placebo	Yes (not stratified)	Indirect	Investigator-assessed DFS	Improved DFS and OS in patients who were ctDNA + and received adjuvant atezolizumab	N/A	Yes	DFS HR: 0.64 (ctDNA + subgroup)	0.005
NIAGARA	Adults with MIBC eligible to receive cisplatin and undergo surgery (1063)	Durvalumab + chemotherapy vs. chemotherapy alone	No	Extrapolative	EFS, OS	Neoadjuvant chemotherapy + durvalumab improved EFS and OS	N/A	No	EFS HR: ~0.68, CI: 0.56–0.82; OS HR: ~0.75, CI: 0.59–0.93	EFS < 0.001; OS *p* = 0.01
CheckMate901	Adults with metastatic bladder cancer (608)	Nivolumab + chemotherapy vs. chemotherapy alone	Yes (~20–25%)	Direct	OS, PFS	Nivolumab + platinum chemotherapy improved OS vs. chemotherapy alone	N/A	No	OS HR: 0.78; PFS HR: 0.72	OS *p* = 0.02; PFS *p* = 0.01
EV 302/KEYNOTE A39	Adults with metastatic bladder cancer (886)	Enfortumab vedotin + pembrolizumab vs. chemotherapy alone	Yes (~25–30%)	Extrapolative	OS, PFS	EV + Pembrolizumab was superior to platinum chemotherapy for OS and PFS	Improvement in OS and PFS in UTUC subgroup	No	OS HR: ~0.47; PFS HR: ~0.45	OS and PFS *p* < 0.0001
EV-303/KEYNOTE905	Adults with MIBC and are cisplatin ineligible but able to undergo surgery (344)	Enfortumab vedotin + pembrolizumab vs. surgery alone	No	Extrapolative	EFS	EV + Pembrolizumab reduced recurrence/progression/death and improved OS (secondary end-point)	N/A	Yes (exploratory)	EFS HR: 0.40	*p* < 0.001 (EFS)
EV-304/KEYNOTEB15	Adults with MIBC and are cisplatin-eligible and able to undergo surgery (808)	Enfortumab vedotin + pembrolizumab vs. chemotherapy alone	No	Extrapolative	EFS	EV + Pembrolizumab was superior to neoadjuvant platinum chemotherapy	N/A	Yes (exploratory)	EFS HR: ~0.53	*p* < 0.0001

(UTUC, upper tract urothelial carcinoma; DFS, disease-free survival; EFS, event-free survival; OS, overall survival; PFS, progression-free survival; ITT, intention to treat).

**Table 2:** UTUC-specific and UTUC-relevant ctDNA/utDNA evidence and ongoing investigational studies, classified by level of directness.

Trial/Study	Trial Population (N)	Study Design/Intervention	ctDNA Assay/Biomarker	Relevance to UTUC	Primary Endpoint(s)	Key Efficacy Outcome(s)	UTUC Specific Outcomes	Hazard Ratio	*p*-Value
Huelster et al. [2]	Adults with UTUC (30)	Utilization of ctDNA to diagnose/risk stratify	Plasma ctDNA	Direct	PFS; CSS	ctDNA positivity correlated to a decrease in PFS and CSS	Prognostication of UTUC	PFS HR: 18.5 CSS HR: 9.3	PFS < 0.001 CSS < 0.016
Nakano et al. [9]	Adults with UTUC (50)	Utilization of ctDNA to prognosticate/drive treatment decisions	Plasma ctDNA	Direct	RFS, OS	Preoperative ctDNA negativity or ctDNA + <2% indicated improved RFS and OS compared to ctDNA+ > 2%	Prognostication utilizing ctDNA in UTUC	RFS HR: 13.75 OS HR: 32.93	RFS = 0.0002 OS < 0.0001
Zhang et al. [10]	Adults with resectable MIBC (20)	Assessment of urine tumor DNA following neoadjuvant immunotherapy	Urine Tumor DNA	Indirect	pCR	Improved pCR in patients with urine tumor DNA response after neoadjuvant therapy	N/A	N/A	N/A
IMvigor010 (correlative analysis)	Adults with MIBC able to undergo surgery and ctDNA+ after surgery	Adjuvant atezolizumab vs. observation alone	ctDNA MRD	Direct	DFS, OS (for ctDNA+ patients after cystectomy)	Patients who were ctDNA+ after cystectomy had worse outcomes than ctDNA- patients	N/A	DFS HR: 6.3, OS HR: 8.0	DFS < 0.0001 OS < 0.0001
IMvigor011	Adults with MIBC and are disease-free radiologically (761)	ctDNA+ patients randomized to receive atezolizumab or placebo	Plasma ctDNA MRD	Indirect	Investigator-assesed DFS	Improved DFS and OS in patients who were ctDNA+ and received adjuvant atezolizumab	N/A	DFS HR: 0.64 (ctDNA + subgroup)	0.005
MODERN	Adults with MIBC who have undergone surgery (ongoing)	ctDNA assessment post-op and randomization into treatment vs. surveillance arms	Plasma ctDNA MRD	indirect	OS, ctDNA clearance (if initially +); DFS (if initially ctDNA −)	Ongoing, prospective trial	N/A	N/A	N/A
TOMBOLA	Adults with MIBC who have undergone radical cystectomy (ongoing)	ctDNA assessment post-op and randomization between surveillance (ctDNA-) or adjuvant immunotherapy (ctDNA+)	Plasma ctDNA MRD	extrapolative	CR (ctDNA clearance and no radiographic evidence of disease)	Ongoing, prospective trial	N/A	N/A	N/A

(OS, overall survival, PFS, progression-free survival; CSS, cause-specific survival; RFS, recurrence-free survival; UTUC, upper tract urothelial carcinoma; pCR, pathologic complete response; DFS, disease-free survival).

**Table 3:** Proposed investigational ctDNA/utDNA-guided framework in UTUC.

Preoperative ctDNA-positive → potential neoadjuvant systemic therapy
Postoperative ctDNA-positive → potential adjuvant systemic therapy
Postoperative ctDNA-negative → potential surveillance or treatment de-escalation
Persistent or rising ctDNA → potential treatment modification or escalation

## Data Availability

No new data were created or analyzed in this study. Data sharing is not applicable to this article.

## Abbreviations

The following abbreviations are used in this manuscript:

UTUC	Upper tract urothelial carcinoma
UC	Urothelial cancer
TURBT	Transurethral resection of bladder tumor
CtDNA	Circulating tumor DNA
MRD	Molecular residual disease
ADC	Antibody–drug conjugate
EV	Enfortumab vedotin
MIUC	Muscle invasive urothelial cancer
UtDNA	Urinary-based tumor DNA
RNU	Radical nephroureterectomy
